# Divergent Characteristics of T-Cell Receptor Repertoire Between Essential Hypertension and Aldosterone-Producing Adenoma

**DOI:** 10.3389/fimmu.2022.853403

**Published:** 2022-05-10

**Authors:** Che-Mai Chang, Kang-Yung Peng, Chieh-Kai Chan, Yu-Feng Lin, Hung-Wei Liao, Jan-Gowth Chang, Mai-Szu Wu, Vin-Cent Wu, Wei-Chiao Chang

**Affiliations:** ^1^Ph.D. Program in Medical Biotechnology, College of Medical Science and Technology, Taipei Medical University, Taipei, Taiwan; ^2^Department of Internal Medicine, National Taiwan University Hospital, Taipei, Taiwan; ^3^TAIPAI, Taiwan Primary Aldosteronism Investigation (TAIPAI) Study Group, Taipei, Taiwan; ^4^Chinru Clinic, Department of Nephrology, Taipei, Taiwan; ^5^Epigenome Research Center, China Medical University Hospital, Taichung, Taiwan; ^6^School of Medicine, China Medical University, Taichung, Taiwan; ^7^Division of Nephrology, Department of Internal Medicine, Taipei Medical University-Shuang Ho Hospital, New Taipei City, Taiwan; ^8^Department of Internal Medicine, School of Medicine, College of Medicine, Taipei Medical University, Taipei, Taiwan; ^9^TMU-Research Center of Urology and Kidney (TMU-RCUK), Taipei Medical University, Taipei, Taiwan; ^10^Division of Nephrology, Department of Internal Medicine, National Taiwan University Hospital, Taipei, Taiwan; ^11^Master Program in Clinical Pharmacogenomics and Pharmacoproteomics, School of Pharmacy, Taipei Medical University, Taipei, Taiwan; ^12^Department of Clinical Pharmacy, School of Pharmacy, Taipei Medical University, Taipei, Taiwan; ^13^Integrative Research Center for Critical Care, Department of Pharmacy, Wanfang Hospital, Taipei Medical University, Taipei, Taiwan; ^14^Department of Medical Research, Taipei Medical University-Shuang Ho Hospital, New Taipei City, Taiwan

**Keywords:** hypertension, aldosterone-producing adenoma (APA), high-throughput sequencing, T-cell receptor (TCR), TCR repertoire, Taiwan Primary Aldosteronism Investigation (TAIPAI)

## Abstract

Aldosterone-producing adenoma (APA) is a benign adrenal tumor that results in persistent hyperaldosteronism. As one major subtype of primary aldosteronism, APA leads to secondary hypertension that is associated with immune dysregulation. However, how the adaptive immune system, particularly the T-cell population, is altered in APA patients remains largely unknown. Here, we performed TCR sequencing to characterize the TCR repertoire between two age-matched groups of patients: one with APA and the other one with essential hypertension (EH). Strikingly, we found a significant reduction of TCR repertoire diversity in the APA group. Analyses on TCR clustering and antigen annotation further showed that the APA group possessed lower diversity in TCR clonotypes with non-common antigen-specific features, compared with the EH group. In addition, our results indicated that the strength of correlation between generation probabilities and frequencies of TCR clonotypes was significantly higher in the APA group than that in the EH group. Finally, we observed that clinical features, including plasma aldosterone level, aldosterone–renin ratio, and blood sodium level, were positively associated with the strength of correlation between generation and abundance of TCR clonotypes in the APA group. Our findings unveiled the correlation between T-cell immune repertoire and APA, suggesting a critical role of such adrenal adenoma in the T-cell immunity of patients with hypertension.

## Introduction

Present in 5%–20% or more of patients with hypertension (HT), primary aldosteronism (PA) is the most common cause of secondary HT (1, 2). PA is characterized by hyperaldosteronism, hypokalemia, high blood pressure, and low plasma renin activity (3). The major causes of PA include aldosterone-producing adenoma (APA) and bilateral adrenal hyperplasia (BAH). While APA is a unilateral benign tumor (non-cancerous proliferation) that develops in one of the adrenal glands, BAH develops at both (2). Such abnormalities lead to sustained overproduction of aldosterone, which promotes salt intake, vasoconstriction, and tissue inflammation. High salt intake and sustained vasoconstriction in turn cause persistent elevation of blood pressure (4). Consistently, patients with PA possess higher cardiovascular morbidity and mortality than those with essential hypertension (EH) (5, 6). The pathogenesis may be attributed to the abnormality of ion channels. In fact, several lines of evidence have shown that genetic disorders were implicated in the development of PA, including somatic mutations on *KCNJ5* and *CACNA1D*, genes that encode potassium and calcium channels, respectively, and are widely found abnormal in APA (7, 8). Other ion channel and cellular signaling transducer genes were also identified to carry somatic mutations in a small proportion of APA patients. Examples include *ATP1A1*, *ATP2B3*, and *CTNNB1* (9–12), with mutated *ATP1A1* and *ATP2B3* shown to increase aldosterone production (13, 14). These findings suggest an important role of somatic mutations in the pathogenesis of PA, particularly APA, through promoting tumor development and/or increasing aldosterone biosynthesis.

In addition to HT and cardiovascular diseases, patients with PA exhibit a higher risk of diabetes and metabolic disorder than those with EH (15). Sustained hyperaldosteronism is also known to increase cardiac and renal damages independent of its high blood pressure level (16, 17). A potential mechanism is the enhancement of tissue inflammation in PA. In fact, hyperaldosteronism has been considered an endocrine disorder that affects the immune system (18, 19). Evidence includes an association of hyperaldosteronism with inflammatory responses and immune dysregulation, such as the generation of oxidative stress and the release of pro-inflammatory cytokines (20, 21). Likewise, systemic infusion of aldosterone leads to tissue inflammation and the production of oxidative stress in rat myocardium (22). In patients with PA or APA, a sustained high blood aldosterone level was found to correlate with the risk of sepsis, likely through chronic inflammation and immune dysfunction (23). In line with this finding is the ability of aldosterone to promote CD8^+^ T-cell activation and CD4^+^ T-cell polarization toward the T helper 17 (Th17) subset by stimulating dendritic cells (DCs) (24). Therefore, an emerging view is that PA and APA are linked to genetic mutations and immune dysregulation. Changes of the immune repertoire have been reported in the response to virus infection, in the progression of kidney diseases, and in the development of tumors (25–29). However, whether PA and APA patients exhibit divergent profiles of T-lymphocyte repertoire remains elusive. In this study, we performed high-throughput sequencing (HTS) to construct TCRβ repertoires in two age-matched groups of patients: one with APA and the other with EH. The differences in the characteristics of TCRβ repertoire between the two groups were investigated. Our results indicated that TCRβ repertoire diversity was significantly different between these two groups. In addition, the diversity of TCRβ clonotypes with predicted non-common antigen specificities in the APA group was distinct from the EH groups. Another result was that the strength of association between frequencies and generation probabilities of TCRβ clonotypes was significantly different, along with a connection of such correlation coefficient with aldosterone, renin, and sodium levels in the APA group. Our findings suggested a critical role of APA in the T-cell immunity of HT patients.

## Materials and Methods

### Data Sources and Study Population

Eight patients aged >18 years who had been diagnosed with PA were recruited. For quality affirmation, the Taiwan Primary Aldosteronism Investigators (TAIPAI) database was used in order to standardize data collection. Furthermore, eight essential hypertensive (EH) patients matched with age were enrolled as control patients. All antihypertensive medications were discontinued for at least 21 days before PA confirmation tests. Doxazosin and/or diltiazem was administered to control markedly high blood pressure during the screening stage when required. The diagnosis of PA in hypertensive patients was based on the inappropriate hypersecretion of aldosterone and according to the fulfillment of the standard criteria (30).

### Confirmation Tests

Fulfillment of the following three conditions confirmed a diagnosis of PA: 1) autonomous excess aldosterone production evidenced with an aldosterone–renin ratio (ARR) >35 (ng/dl)/(ng/ml/h), 2) a TAIPAI score larger than 60%, and 3) post-saline loading of plasma aldosterone concentration (PAC) >16 ng/dl or PAC/plasma renin activity (PRA) ratio >35 (ng/dl)/(ng/ml/h) shown in a post-captopril/losartan test (31).

APA was identified on the basis of the following four criteria (31): 1) confirmed PA; 2) an adrenal adenoma or hyperplasia evidenced with a CT or MRI scan; 3) lateralization of aldosterone secretion with adrenal vein sampling (AVS) or during dexamethasone suppression NP-59 SPECT/CT on the imaging finding side, which is further confirmed after adrenalectomy; and 4) pathologically proven CYP11B2 adenoma or aldosterone-producing cell clusters at immunohistochemistry after adrenalectomy and the subsequent emergence of biochemical correction.

### Ethical Approval of the Study Protocol

The study complied with the Declaration of Helsinki and was approved by the National Taiwan University Hospital Research Ethics Committee (No. 200611031R). All participants received comprehensive written information and signed a consent form before their inclusion in the study.

### Sample Preparation

Whole blood samples were collected from APA and EH patients and subjected to peripheral blood mononuclear cell (PBMC) isolation using a density-gradient separation approach. Total RNA was extracted from PBMCs using a column-based method with RNeasy Mini Kit (Qiagen, Hilden, Germany). RNA quality was assessed by NanoDrop (Thermo Fisher Scientific, MA, USA) and Qsep100 (BiOptic, New Taipei City, Taiwan) to ensure that the yield of RNAs was sufficient for TCR sequencing analysis.

### TCR Library Preparation and Sequencing

The preparation of TCR libraries for TCR sequencing was performed as previously described (29, 32). In brief, cDNA was synthesized from extracted RNA using the Switching Mechanism At 5′ end of RNA Template (SMART) technology with SMARTer PCR cDNA Synthesis Kit (Takara Bio USA, CA, USA) and a self-modified cDNA synthesis (CDS) oligo (5′-CGG GGT ACG ATG AGA CAC CAT TTT TTT TTT TTT TTT TTT TVN-3′). The full-length cDNA was amplified using Q5 High-Fidelity 2X Master Mix (New England Biolabs, MA, USA) with the 5′ PCR primer II A (Takara Bio USA, CA, USA) and a customized CDS primer. To enrich the VDJ regions of TCRβ cDNA, the 5′ Rapid Amplification of cDNA Ends (5′ RACE) technology was employed using Q5 High-Fidelity 2X Master Mix with customized template-switch oligo (TSO) and TRBC1/TRBC2-specific primers. Following the cDNA enrichment, PCR products were subjected to the size selection step to capture enriched TCRβ cDNA fragments within the size range of 300~1,000 bp by the Pippin DNA Size Selection System (Sage Science, MA, USA). Illumina index and adaptor sequences were subsequently introduced to TCRβ fragments using KAPA HiFi HotStart ReadyMix (Roche, Basel, Switzerland) with Nextera XT Index Kit (Illumina, CA, USA). Finally, all TCRβ libraries were pooled and sequenced using the Illumina MiSeq platform with MiSeq Reagent Kit v3 (2 × 300 bp read length). For every cDNA amplification and size selection step, PCR products were purified using AMPure XP reagent kit (Beckman Coulter, CA, USA). The quantification of the TCRβ library was performed using KAPA Library Quantification Kit (Roche, Basel, Switzerland).

### TCR Repertoire Data Collection and Analysis

#### Sequencing Data Preprocessing

The sequencing raw data generated in a FASTQ format were subjected to trimming, filtering, alignment, and assembly processes as previously described (29, 32). Firstly, TSO and Illumina adaptor sequences were removed, respectively, from the 5′ and 3′ ends of raw sequencing reads using Cutadapt (version 3.0). The quality filtering was then performed to remove low-quality bases/reads using Trimmomatic (version 0.39) with self-defined parameters (LEADING: 15, TRAILING: 15, SLIDINGWINDOW: 4:20, MINLEN: 50). After filtering, MiXCR (version 3.0.12) was employed to align these reads to variable (V), diversity (D), and joining (J) gene segments of the TCRβ gene and to further assemble TCRβ clonotypes based on CDR3 nucleic acid sequences of aligned reads; accordingly, a table containing the CDR3 sequence, clonal abundance, and V(D)J alleles of each TCRβ clonotype was generated as the TCRβ repertoire profile of each subject. The clonotype table was subsequently collapsed by merging TCR clonotypes with identical CDR3 amino acid sequence. For each new TCRβ clonotype, the clonal abundance was determined by accumulating counts of distinct CDR3 nucleic acid sequences corresponding to the same CDR3 amino acid sequence, and the V(D)J alleles of dominant CDR3 nucleic acid sequences with the highest counts were used as representative.

#### Calculation of TCR Repertoire Diversity and Similarity

The diversity profile of the TCRβ repertoire was based on Hill’s numbers (*^α^D*) calculated as follows:

$$\alpha D={\left(\sum_{i=1}^{N}p_{i}^{\alpha }\right)}^{\frac{1}{1-\alpha }}$$

where *N* is the number of unique TCRβ clonotypes of each TCRβ repertoire and *p_i_* is the frequency of the *i*^th^ clonotype. The *α* value indicates weights on frequencies of each clonotype, and abundant clonotypes are weighted more along with the increase of the *α* value. When *α* = 0, *^α^D* is the richness of the TCRβ repertoire, whereas *^α^D* represents the dominance of abundant TCRβ clonotypes when *α* >0. In the study, *^α^D* was computed in the range of *α* from 0 to 10. Since the *^α^D* number is undefined when *α* = 1, the exponential of Shannon entropy (*H′*) is calculated instead. For data visualization, *^α^D* was transformed to Rényi entropy (*^α^H*) by natural logarithm as follows:


$$\alpha H=I\text{n}{(}^{\alpha }D)$$

The mean of transformed values *^α^H* was computed for each patient group. The continuous diversity profile was generated using the *loess()* function of *stats* R package, which is based on the locally estimated scatterplot smoothing (LOESS) method.

For the comparison of diversity indices of TCRβ repertoires, Pielou’s evenness index (*J′*), which takes the maximum *H′* into account, was calculated as follows:

$$J^{\prime }=\frac{H^{\prime }}{{H^{\prime }}_{\text{max}}}=\frac{-\sum_{i=1}^{N}p_{i}\times \text{In}(p_{i})}{\text{In}(N)}$$

where *N* is the number of unique TCRβ clonotypes and *p_i_* is the frequency of the *i*^th^ clonotype in the TCRβ repertoire.

To evaluate the TCRβ repertoire similarity among patients, the Morisita–Horn (MH) index was calculated as follows:

$$\text{MH}=\frac{2\times \sum_{i=1}^{N}x_{i}y_{i}}{\sum_{i=1}^{N}\left(x_{i}^{2}+y_{i}^{2}\right)}$$

where *N* is the total number of unique TCRβ clonotypes from sample X plus sample Y, and *x_i_* and *y_i_* are the frequencies of the *i*^th^ clonotype in sample X and sample Y, respectively. The value of the MH index, ranging from 0 (no overlap) to 1 (identical), represents the extent of similarity between two TCRβ repertoires.

#### Assessment of V/J Gene Usage, V–J Gene Pairing, and CDR3 Length Profiles

Profiling of V/J gene usage, V–J gene pairing, and CDR3 length distribution of each TCRβ repertoire was performed by calculating the occurrences of each TRBV/TRBJ allele, each V–J segment pair, and each CDR3 length in unique TCRβ clonotypes, respectively. The repertoire dissimilarity index (RDI) was calculated to quantify the similarity in V/J gene usage, V–J gene pairing, and CDR3 length between any two of patients as previously described (33). The RDI analysis was performed using the *rdi()* function of *rdi* R package.

#### Integration of TCR Clustering and Annotation

The TCR clustering was performed using the Grouping of Lymphocyte Interaction by Paratope Hotspots version 2 (GLIPH2, version 0.01) with recommended parameters (http://50.255.35.37:8080/) to identify TCRβ clusters, where clustered TCRβ clonotypes had a high possibility of sharing antigen specificity, for each patient’s TCRβ repertoire (34). Based on the global similarity and motif enrichment of CDR3 sequences of clustered TCRβ clonotypes, each TCRβ cluster was classified as global-based, motif-based, or mixed type (35). On the basis of the results by GLIPH2, those of the TCRβ clonotypes that were not grouped were defined as non-clustered clonotypes.

For TCR annotation, the information of human TCRβ clonotypes collected in the VDJdb (version 2020-05-20) database was downloaded (https://github.com/antigenomics/vdjdb-db) as a reference of common antigen-specific TCRs (36). In the study, we used two strategies to annotate TCRβ clonotypes with possible common antigen specificities as follows:

1. The V gene and CDR3 sequence of each clustered or non-clustered TCRβ clonotype from our study cohort were matched directly against those from VDJdb by VDJmatch (version 1.3.1) software. The query TCRβ clonotype was considered as a common antigen-specific TCR when its V gene and CDR3 sequence were identical to that of any TCRβ clonotype from VDJdb.
2. TCRβ clonotypes from VDJdb were firstly grouped by their target epitopes (antigens). Each group of VDJdb TCRβ clonotypes was then subjected to TCR clustering using GLIPH2 as described above; accordingly, common antigen-specific TCRβ clusters were identified and classified as global-based and/or motif-based type(s). In the TCR annotation step, we first mapped both of our clustered and non-clustered TCRβ clonotypes to VDJdb clonotypes of global-based clusters using VDJmatch. The query TCRβ clonotype that had the same V gene and highly similar CDR3 sequence (≤1 mismatch) as any of global-clustered common antigen-specific VDJdb clonotypes was considered as a common antigen-specific TCR. Next, we matched enriched CDR3 motif sequences of our motif-based clusters against those of VDJdb motif-based clusters. When the sequence of CDR3 motif enriched at query motif-based cluster was found to match one at VDJdb motif-based clusters, all clonotypes of the motif-based cluster were considered to possess common antigen specificity/specificities.

TCRβ clonotypes that were able to pass any of the matching/mapping rules described above were annotated as common antigen-specific clonotypes. TCRβ clusters with at least one common antigen-specific TCRβ clonotypes were defined as common antigen-specific clusters. Accordingly, TCRβ clonotypes either grouped into common antigen-specific clusters or annotated as non-clustered common antigen-specific clonotypes were assigned to common antigen-specific features, whereas the remaining clustered/non-clustered TCRβ clonotypes were considered to possess non-common antigen-specific features.

#### Evaluation of the Correlation Between Clonal Frequency and Generation Probability (*P*_gen_)

To estimate the generation probability (*P*_gen_) of each TCRβ clonotype in our study cohort, the Optimized Likelihood estimate of immunoGlobulin Amino-acid sequences (OLGA, version 1.2.1) was adopted (37). The *P*_gen_ of each TCRβ clonotype was computed on the basis of its CDR3 amino acid sequence. To assess the correlation between clonal frequencies and *P*_gen_ for each sample, log10-transformed values of frequencies and *P*_gen_ of TCRβ clonotypes were calculated prior to the correlation test and comparison.

### Statistical Analysis

All statistical analyses of the study were performed using R software (version 3.6.1). The data visualization was carried out using *ggplot2* and *ggpubr* R packages. The comparison of repertoire diversity, clonal abundance, and correlation between clonal frequency and *P*_gen_ between different patient groups was performed using the two-sided Wilcoxon signed-rank test (paired samples). The comparison of MH and RDI was performed using the two-sided Wilcoxon rank-sum test. The Pearson correlation coefficient (Pearson’s *r*) was calculated to assess the linear correlation between clonal frequencies and *P*_gen_ of each subject’s repertoire. The association between clinical measurements and the correlation coefficient between clonal frequency and *P*_gen_ was evaluated using linear regression analysis. *p*-values were corrected by the false discovery rate (FDR) using the *p.adjust()* function of *stats* R package. The statistical significance was denoted by “*” (adjusted *p*-value < 0.05), “**” (adjusted *p*-value < 0.01), or “***” (adjusted *p*-value < 0.001).

## Results

### Baseline Characteristics

We enrolled eight patients with newly identified APA (women, 25.0%; mean, 47.7 years) and eight age-matched patients with EH (women, 37.5%; mean, 49.3 years) as controls (**Table 1**). The difference of age in each pair between the APA and matched EH patients was less than 5 years. APA patients exhibited higher PAC, higher ARR, lower PAC/PRA, and lower serum potassium than EH patients.

**Table 1:** Summary statistics of essential hypertension (EH) and aldosterone-producing adenoma (APA) patients.

	EH (*n* = 8)	APA (*n* = 8)	*p*-value^a^
Age (years), mean (SD)	49.3 (11.2)	47.7 (10.6)	0.574
Gender, *N* (%)			
Male	5 (62.5)	6 (75.0)	1.000
Female	3 (37.5)	2 (25.0)	
Blood pressure (mmHg), mean (SD)			
Systolic blood pressure	143.6 (19.5)	165.5 (29.3)	0.172
Diastolic blood pressure	91 (12.2)	101.5 (13.3)	0.172
Diabetes mellitus, *N* (%)	1 (12.5)	2 (25.0)	1.000
Hypertension, *N* (%)	8 (100.0)	8 (100.0)	1.000
Plasma aldosterone (ng/dL), mean (SD)	20.2 (13.1)	228.4 (173.7)	0.007^**^
Plasma renin activity (ng/mL/h), mean (SD)	2.9 (2.4)	1.1 (1.8)	0.024^*^
Potassium (mmol/L), mean (SD)	4.1 (0.3)	3.5 (0.4)	0.020^*^

a p-values for continuous and categorical data were shown from the Wilcoxon rank-sum test and Fisher’s exact test, respectively. P-values <0.05 or <0.01 were considered to be statistically significant and marked with “*” or “**”, respectively.

### Distinct Characteristics of the TCRβ Repertoire Between APA and EH Patients

**Figure 1:**
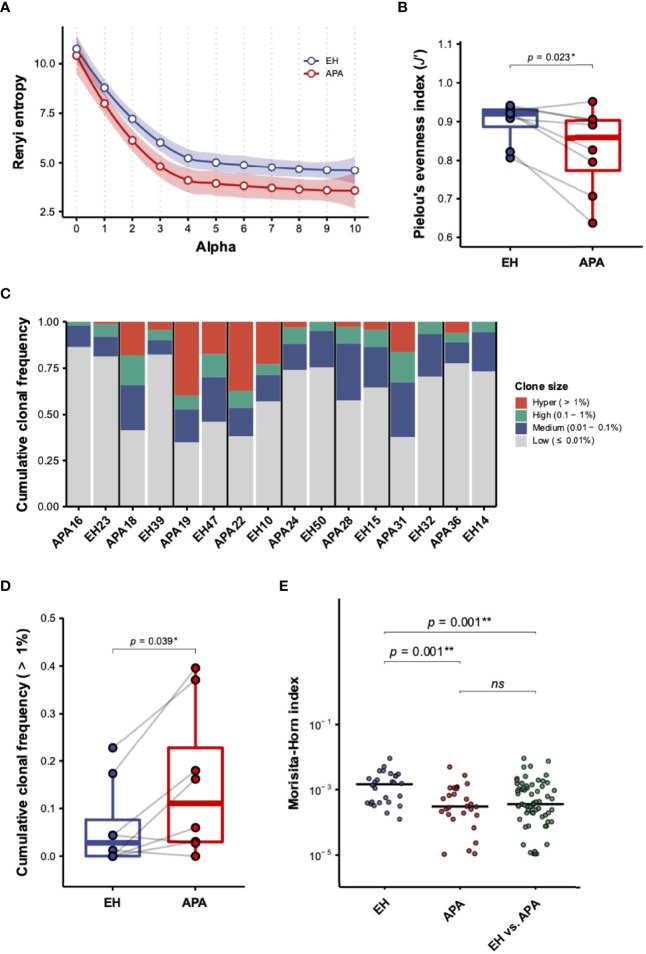
The characterization of TCRβ repertoire in aldosterone-producing adenoma (APA) and essential hypertension (EH) patients. **(A)** The diversity profile based on the Rényi entropy (*^α^H*) was illustrated. Each dot represented the mean of Rényi entropies of EH (blue dot) or APA (red dot) patients for a specific order of *α* (alpha) value. The smooth line and shading region indicated the estimated local polynomial regression fitting curve and 95% confidence intervals (CIs), respectively, of Rényi entropies in the EH (blue color) and APA (red color) groups with *α* value ranging from 0 (clonal richness) to 10 (clonal dominance). The local regression was performed using the locally estimated scatterplot smoothing (LOESS) method. **(B)** The difference in repertoire diversity was evaluated by comparing Pielou’s evenness indices between APA and age-matched EH patients. The solid gray line denoted age-matched pairs. A two-sided *p*-value was shown from the Wilcoxon signed-rank test. **(C)** Cumulative frequencies of TCRβ clonotypes of hyper- (>1%), high- (0.1%~1%), medium- (0.01%~0.1%), and low-abundant (≤0.01%) clone sizes were illustrated for each patient. Stacked bars with cumulation of clonal frequencies for different clone sizes of each pair of APA patients and age-matched EH individuals were displayed. **(D)** The cumulative frequency of hyper-abundant (>1%) TCRβ clonotypes was compared between APA and age-matched EH patients. The solid gray line denoted age-matched pairs. A two-sided *p*-value was shown from the Wilcoxon signed-rank test. **(E)** The Morisita–Horn (MH) index was calculated for intragroup (any two of APA or EH patients) and intergroup (any one of EH vs. one of the APA patients) repertoire similarity. The comparison between intragroup and intergroup MH indices was depicted. The horizontal line represented the median. The FDR-adjusted two-sided *p*-values were shown from the Wilcoxon rank-sum test. The results of the comparison with corrected *p*-value >0.05 were considered to be not statistically significant and denoted by “ns” (not significant). *p*-values < 0.05 or < 0.01 were considered to be statistically significant and marked with ‘*’ or ‘**’, respectively.

We isolated PBMCs from APA patients and age-matched EH individuals for TCRβ repertoire sequencing. We first assessed the difference in TCRβ repertoire diversity between APA and EH patients. The diversity profiles by Rényi entropy revealed that both richness (*α* = 0) and dominance (*α* > 0) of the TCRβ repertoire were much lower in APA patients than in EH patients (**Figure 1A**). The comparison of Pielou’s evenness index further confirmed a significant decrease of TCRβ repertoire diversity in APA patients (**Figure 1B**). Such reduction of TCRβ repertoire diversity in the APA group suggested that APA patients experienced TCRβ clonal expansion. Indeed, most of the APA patients had a larger proportion of abundant TCRβ clonotypes compared with the age-matched EH individuals (**Figure 1C**). The cumulative frequency of hyperexpanded TCRβ clonotypes, defined as those with clone size larger than 1%, was significantly higher in APA than in age-matched EH patients (**Figure 1D**). Similar tendencies toward increased clonal frequencies of high- (0.1%~1%) and medium-expanded (0.01%~0.1%) TCRβ clonotypes were also observed in APA patients (**Figure S1**).

Following the comparison of repertoire diversity, we evaluated whether TCRβ repertoire similarity based on the MH index between APA patients was divergent within the two groups (**Figure S2**). Our results showed that the MH similarity between TCRβ repertoires of APA patients was significantly lower than that of EH individuals (**Figure 1E**). In addition, the intergroup MH indices between APA and EH patients were also significantly decreased, relative to intragroup MH scores between EH patients. These data suggested that TCRβ repertoires of APA patients were less similar between individuals if compared with EH patients.

To investigate whether characteristics other than repertoire diversity and similarity were skewed in APA, variable (*TRBV*), joining (*TRBJ*) gene usage, and the length distribution of complementarity-determining region (CDR3) were investigated between APA and EH patients. However, neither V nor J gene usage or CDR3 length distribution of the TCRβ repertoire showed a significant difference between APA and EH patients (**Figure S3**). For further validation, we applied the RDI (33) for V/J gene usage, V–J gene pairing, and CDR3 length usage to quantify and compare these characteristics of TCRβ repertoires between APA and EH patients. Similar to our findings above, no significant difference in RDI value was found (**Figure S4**). Thus, our results indicated that V/J gene usage and CDR3 length of the TCRβ repertoire in APA patients were not significantly divergent from those in EH patients. In contrast, the overall TCRβ repertoire diversity and similarity were significantly different between two groups.

### Differential Features of Antigen-Associated TCRβ Clonotypes Between APA and EH Patients

**Figure 2:**
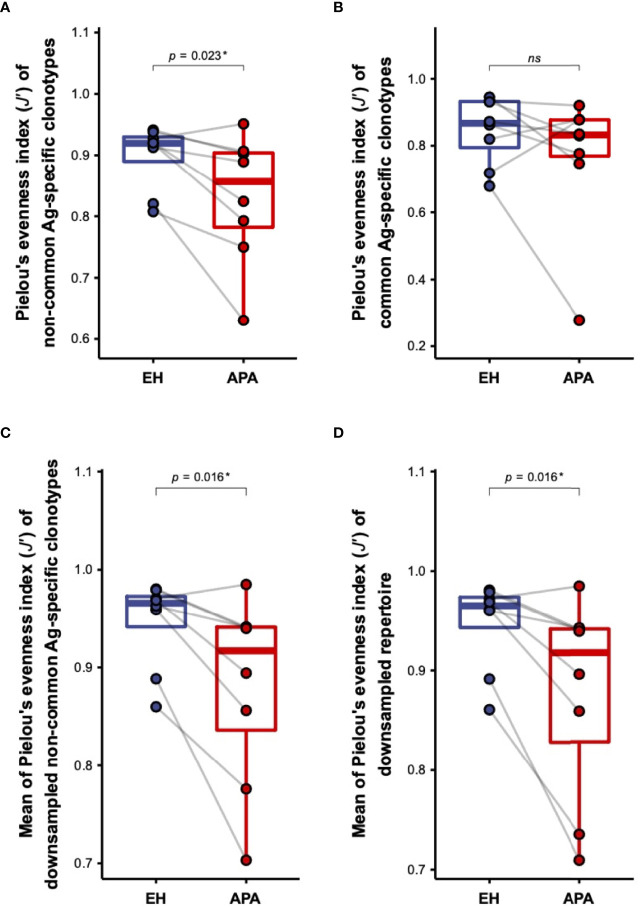
The comparison of the diversity of TCRβ clonotypes with distinct antigen specificities between APA and EH patients. **(A, B)** The difference in repertoire diversity, estimated by the Pielou’s evenness index (*J′*), of non-common antigen-specific **(A)** or common antigen-specific **(B)** TCRβ clonotypes was compared between APA and age-matched EH patients. **(C, D)** The comparison of the mean of diversities *J′* of 100 randomly subsampled TCRβ repertoires from non-common antigen-specific **(C)** or all **(D)** TCRβ clonotypes between APA and age-matched EH patients was illustrated. The solid gray line denoted age-matched pairs and the two-sided *p*-values were shown from the Wilcoxon signed-rank test. The results of the comparison with corrected *p*-value >0.05 were considered to be not statistically significant and denoted by “ns” (not significant). *p*-value < 0.05 was considered to be statistically significant and marked with ‘*’.

According to the difference in overall TCRβ repertoire diversity between APA and EH patients, we speculated that the decrease of TCRβ diversity in APA patients was due to the APA-associated antigen-dependent T-cell response. We thus compared the distinction in repertoire diversity of common antigen-/non-common antigen-specific TCRβ clonotypes between the APA and EH groups. Importantly, we found that the diversity of non-common antigen-specific TCRβ clonotypes was significantly lower in patients with APA than those with EH, whereas there was no significant difference in the diversity of common antigen-specific clonotypes between the two groups (**Figures 2A, B****)**. It is noteworthy that most of the TCRβ clonotypes were assigned to non-common antigen-specific features because of the limited size of the repertoire database for TCR annotation. Such an issue raised a potential concern that the non-significant difference observed in the repertoire diversity of common antigen-specific TCR clonotypes between APA and EH patients might result from the limited number of TCRs annotated with common antigen-specific features. We thus randomly generated 100 subsamples of non-common antigen-specific TCRβ clonotypes with the size equal to common antigen-specific clonotypes for each patient and compared the mean of repertoire diversities of subsampled clonotypes. Similar results showed that the average diversities of subsampled non-common antigen-specific TCRβ clonotypes of APA patients were significantly lower than those of EH individuals (**Figure 2C**). In addition, subsampling analysis for all clonotypes further indicated the lower diversity of subsampled clonotypes in the APA group (**Figure 2D**). These findings indicated that the decrease of TCRβ repertoire diversity in the APA group was contributed by the clonal expansion of TCRβ clonotypes (unrelated to common antigen specificities). The part of these clonotypes with unknown antigen recognition should be associated with APA.

### Divergent Status of the TCRβ Repertoire Driven by Bystander Activation in APA Patients

**Figure 3:**
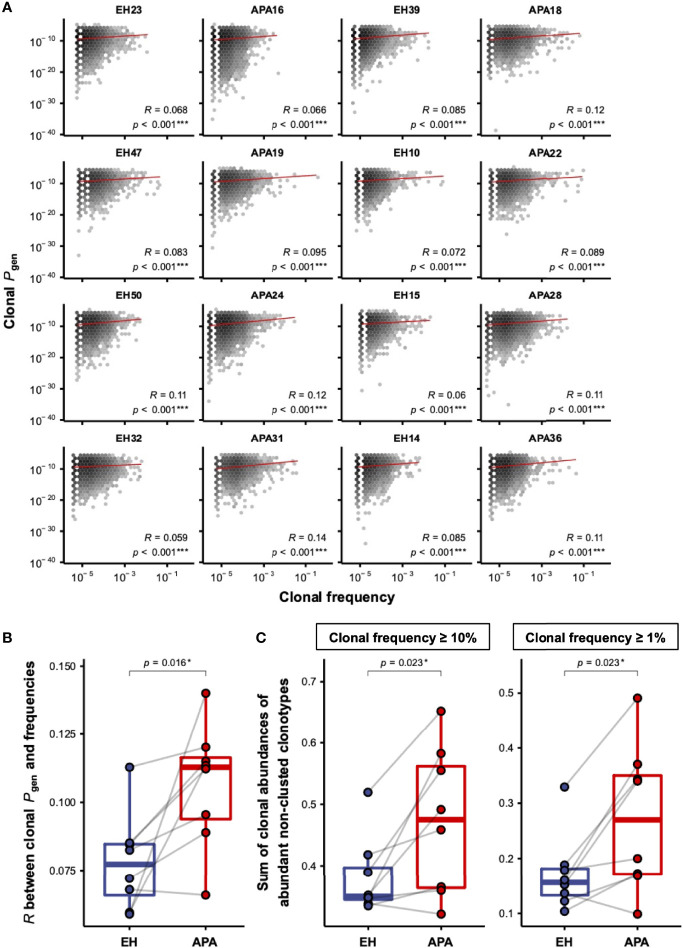
The correlation between frequencies and *P*_gen_ of TCRβ clonotypes in APA and EH patients. **(A)** The distribution of frequencies and *P*_gen_ of TCRβ clonotypes was illustrated by the hexagonal heat map for each patient. The color depth represented the number of TCRβ clonotypes with the corresponding clonal frequency and *P*_gen_. The correlation between log10-transformed frequencies and *P*_gen_ of all clonotypes was computed using linear regression analysis. The solid red line represented the fitted linear regression line for the observed data. The correlation coefficient (*R*) and *p*-value (*p*) of the association between clonal frequencies and *P*_gen_ were labeled for each patient. The two-sided *p*-values were shown from the Pearson correlation test. **(B)** Pearson correlation coefficients (*R*) between log10-transformed frequencies and *P*_gen_ of TCRβ clonotypes were compared between APA and age-matched EH patients. The solid gray line denoted age-matched pairs. The two-sided *p*-values were shown from the Wilcoxon signed-rank test. **(C)** The comparison of cumulative frequencies of abundant TCRβ clonotypes, defined as clonotypes with frequencies larger than or equal to the frequency of clonotype ranked at the top 10% (left panel) or 1% (right panel) of each patient, between APA and age-matched EH patients. The solid gray line denoted age-matched pairs. The two-sided *p*-values were shown from the Wilcoxon signed-rank test. *p*-values < 0.05 or < 0.001 were considered to be statistically significant and marked with ‘*’ or ‘***’, respectively.

Since the effects of aldosterone in T-cell activation have been reported (18), we hypothesized that APA patients may experience hyperaldosteronism-related bystander activation of T cells, which is reflected in the patients’ TCRβ repertoire. On the basis of such speculation, we next estimated the generation probabilities (*P*_gen_) of CDR3 sequences for each TCRβ clonotype using OLGA (37). The correlation between frequencies and *P*_gen_ of TCRβ clonotypes in each APA and EH patient was evaluated. Interestingly, linear regression analysis showed that clonal frequency was significantly correlated with clonal *P*_gen_ in all patients’ TCRβ repertoires (**Figure 3A**). Further comparison of the two groups revealed that Pearson correlation coefficient (Pearson’s *r*) between TCRβ clonal frequencies and *P*_gen_ was significantly higher in APA patients than in age-matched EH individuals (**Figure 3B**). The higher strength of correlation between TCRβ frequency and *P*_gen_ suggested a bystander T-cell activation. We further investigated whether bystander activation was involved in APA, and compared the abundance of non-clustered TCRβ clonotypes, which might be independent from known/unknown antigen-related T-cell response at the time of TCR repertoire sampling between EH and APA patients. Our results indicated significantly higher cumulative frequencies of abundant non-clustered clonotypes in APA patients suggesting that the TCRβ repertoire space of patients with APA was predominantly occupied by non-clustered TCRβ clonotypes among abundant TCRs (**Figure 3C**). Taken together, these lines of evidence revealed the occurrence of bystander T-cell activation in APA patients which performed a stronger strength of association between TCRβ abundance and generation, and possessed a higher proportion of abundant non-clustered TCRβ clonotypes.

### Correlation of Clinical Features With TCRβ Repertoire Characteristics in APA and EH Patients

**Figure 4:**
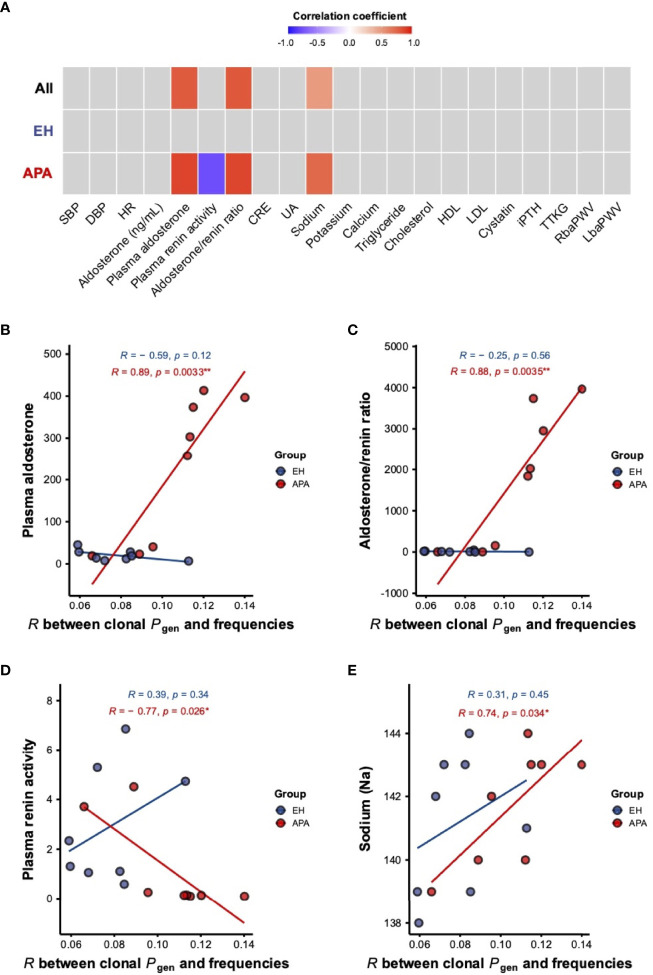
The association of clinical measurements with the strength of correlation between frequencies and *P*_gen_ of TCRβ clonotypes in APA and EH patients. **(A)** Pearson correlation coefficients for the associations of different clinical features with the strength of the correlation between clonal frequencies and *P*_gen_ in all patients and the APA and EH groups were shown in a heat map. Associations with *p*-values <0.05 were considered to be statistically significant and colored either in red (positive) or blue (negative). **(B–E)** Linear correlations of PAC **(B)**, ARR **(C)**, PRA **(D)**, and blood sodium level **(E)** with the correlation coefficient between frequencies and *P*_gen_ of TCRβ clonotypes were illustrated. Pearson’s *r* (*R*) and *p*-value (*p*) of the association were computed for APA (red) and EH (blue) patients. *p*-values <0.05 or <0.01 were considered to be statistically significant and marked with “*” or “**”, respectively. SBP, systolic blood pressure; DBP, diastolic blood pressure; HR, heart rate; CRE, creatinine; UA, uric acid; HDL, high-density lipoprotein; LDL, low-density lipoprotein; iPTH, intact parathyroid hormone; TTKG, transtubular potassium gradient; RbaPWV, right brachial-ankle pulse wave velocity; LbaPWV, left brachial-ankle pulse wave velocity.

We next investigated whether the bystander activation for the T-cell repertoire was associated with clinical/biochemical features in the two groups of patients. As shown in **Figure 4A**, plasma aldosterone concentration (PAC), aldosterone-renin ratio (ARR), and blood sodium level were significantly and positively correlated with the strength of correlation between TCRβ frequencies and *P*_gen_. Particularly, both PAC and ARR were positively correlated with the strength of correlation between clonal frequencies and *P*_gen_ in the APA group, while the association was not observed in the EH group (**Figures 4A–C**). Importantly, we only found that PRA was negatively associated with the strength of correlation between clonal frequencies and *P*_gen_ in the APA group (**Figures 4A, D****)**. Moreover, the blood sodium level was positively correlated with the strength of association between TCRβ frequencies and *P*_gen_ in the APA group but not in the EH group (**Figures 4A, E****)**. These findings implied the connection between APA-related clinical features (including aldosterone, renin, and sodium levels) and TCRβ repertoire status.

## Discussion

In the study, we analyzed the characteristics of the TCRβ repertoire between APA and age-matched EH patients. Results showed that the whole TCRβ repertoire diversity was significantly lower in the APA patient group. By using TCR clustering and antigen annotation approaches, we further determined the TCRβ clonotypes with predicted specificities against non-common or common antigens for each patient. We found that TCRβ clonotypes with non-common antigen-specific features exhibited divergent patterns of TCR diversity between APA and EH patients, whereas the diversity of those with common antigen-specific features in patients with APA was similar to EH individuals. Importantly, the strengths of the association between TCRβ generation probabilities (*P*_gen_) and frequencies in APA patients were significantly higher than that in EH individuals. Taken together, we observed a divergent status of T-cell immunity reflected by the skew of repertoire diversity and by the change of the clonal *P*_gen_ and frequencies in APA patients but not in EH individuals. Finally, our results showed that aldosterone, renin, and sodium levels were significantly and positively associated with the strength of correlation between clonal abundance and generation of TCRβ clonotypes in patients with APA, suggesting the connection between disease activity and T-cell repertoire status in these patients.

Krysiak et al. reported that the levels of tumor necrosis factor-alpha (TNF-α), interleukin 6 (IL-6), and interleukin 1 beta (IL-1β) from monocytes/macrophage and interleukin 2 (IL-2), interferon-gamma (IFN-γ), and TNF-α from lymphocytes were much higher in patients with aldosteronoma than those in EH individuals, suggesting an active T-cell immunity in APA patients (38). Additionally, it has been known that most of the APA patients harbor KCNJ5 somatic mutations, especially in Asian populations (39, 40). Consistent with these findings, our data support such ideas that the diversity of non-common antigen-specific clonotypes was significantly different between the APA and EH groups. The reduction of TCR repertoire diversity in the APA group might be attributed to the peripheral T-cell clonal expansion which was probably elicited by tumor immunogenicity of adrenal adenoma through adenomatous neoantigens. Indeed, the correlation between premalignant neoantigens and infiltration of T lymphocytes was nicely reported in lung adenomatous hyperplasia (41). However, whether the neoantigens were generated by somatic mutations in APA and subsequently induced T-cell response through TCR recognition still needs further investigation.

Previous studies have reported that adaptive immune cells of APA patients were affected by abnormal aldosterone secretion (18). Aldosterone is capable of promoting activation for CD8^+^ T cells and Th17 polarization of CD4^+^ T cells through stimulation of DCs (24). In addition, aldosterone was found to reduce the number of regulatory T cells (Tregs), a T-cell subpopulation that negatively modulates the immune response, in the kidney (42). These lines of evidence suggested that adenoma-induced hyperaldosteronism might result in T-cell expansion. Therefore, the decrease of T-cell repertoire diversity observed in APA patients may be due to aldosterone-related bystander activation of T cells through cytokine stimulation. Such hyperaldosteronism-related bystander T-cell activation may cause antigen-independent expansion of the T-cell repertoire, and as a result, the TCR clonotypes are easier to be generated *via* the VDJ recombination (high clonal *P*_gen_) and to be more abundant (high clonal frequency). In light of this, bystander T-cell activation/proliferation might provide a mechanistic explanation for the frequencies and *P*_gen_ of TCRβ clonotypes being significantly higher under hyperproduction of aldosterone in APA patients. Such mechanisms could explain why we observed that PAC and ARR were positively associated with the strength of correlation between the frequencies and *P*_gen_ of TCRβ clonotypes in the APA group. Our results implied that aldosterone production leads to a higher extent of bystander T-cell activation, which was reflected by a higher strength of correlation between TCR abundances and generation probabilities and APA disease activity.

There are limitations in the study. First, the sample size of our study cohort is small, although this is an age-matched case–control study, which considers the effects of age as a well-known confounder on TCR repertoire status. The small sample size may introduce potential selection bias of samples. Furthermore, there is a lack of information on human leukocyte antigen (HLA) that might influence the TCR annotation for each subject. Inclusion of HLA typing data should enhance the accuracy when matching query CDR3 sequences of TCR clonotypes against those with known antigen specificity and HLA binding from public TCR databases. Moreover, our study did not further characterize the TCR repertoire of different T-cell subsets. Interrogating differences in TCR repertoire characteristics of CD8^+^, Th17, and Treg cell compartment between APA and EH patients would provide advanced information to identify which types of T cells are associated with APA. Finally, our study is restricted by the lack of tumor tissues from APA patients. Profiling of somatic mutations and TCR repertoire in APA tissues will further enable us to identify APA-specific TCR clonotypes and understand how hyperaldosteronism affects the adaptive immune system.

In conclusion, this study discovered the distinct characteristics of the TCR repertoire between essential hypertension and aldosterone-producing adenoma. The results further suggested the importance of antigen-specific TCRβ and bystander T-cell activation in the pathology of adrenal adenoma. Our findings herein provided evidence of the connection between adaptive immune repertoire and APA. The profiling of the immune repertoire could thus be used as a rational strategy that is capable of distinguishing different hypertension subtypes. The application of this approach will accelerate the development of molecular diagnosis for APA in the future.

## Data Availability Statement

The original contributions presented in the study are publicly available. These data can be found at NCBI, under accession number PRJNA798201.

## Ethics Statement

The studies involving human participants were reviewed and approved by the National Taiwan University Hospital Research Ethics Committee (No. 200611031R). The patients/participants provided their written informed consent to participate in this study.

## Author Contributions

V-CW and W-CC initiated the research idea and designed the study. K-YP, C-KC, Y-FL, and H-WL collected the clinical samples and data for the study. C-MC analyzed the data. C-MC, K-YP, J-GC, M-SW, V-CW, and W-CC interpreted the data and results. C-MC, K-YP, V-CW, and W-CC wrote the manuscript. V-CW and W-CC supervised the study. All authors contributed to the article and approved the submitted version.

## Funding

This work was supported by grants from the Ministry of Science and Technology, Taiwan (106-2314-B-002-166-MY3, 107-2314-B-002-026-MY3, 106-2321-B-182-002, 105-2628-B-038-001-MY4, 109-2628-B-038-012, MOST 110-2321-B-038-002 - Establishing A Translational Female Cancer Biomedical Big Data Bank and Developing Precision Medicine Healthcare System), and National Taiwan University Hospital (105-S3061, 107-S3809, UN103-082, UN106-014, 105-P05, 106-P02, 107-T02).

## Conflict of Interest

The authors declare that the research was conducted in the absence of any commercial or financial relationships that could be construed as a potential conflict of interest.

## Publisher’s Note

All claims expressed in this article are solely those of the authors and do not necessarily represent those of their affiliated organizations, or those of the publisher, the editors and the reviewers. Any product that may be evaluated in this article, or claim that may be made by its manufacturer, is not guaranteed or endorsed by the publisher.
