# Supplementary material for: Divergent Characteristics of T-Cell Receptor Repertoire Between Essential Hypertension and Aldosterone-Producing Adenoma
Source: Front Immunol. 2022 May 10;13:853403. doi: 10.3389/fimmu.2022.853403 (PMC9127864; doi:10.3389/fimmu.2022.853403)
Supplement: Supplementary file 1 [file DataSheet_1.docx]

Supplementary Material

## Supplementary Figures


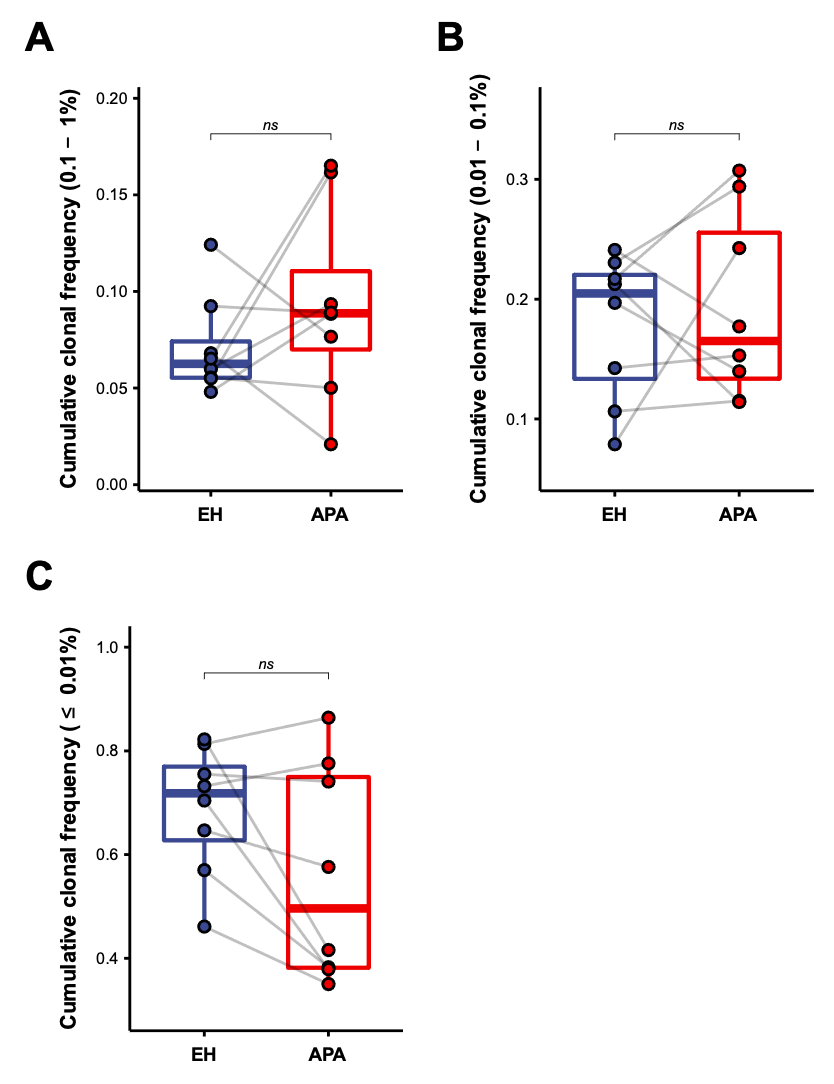


**Figure S1.** The clonal frequency of TCRβ clonotypes of different clone sizes in APA and EH patients. The cumulative frequencies of TCRβ clonotypes of high- (**A**), medium- (**B**) and low- (**C**) abundant clone sizes were compared between APA and age-matched EH patients. The solid gray line denoted age-matched pairs. The two-sided *p*-values were shown from the Wilcoxon signed-rank test. The results of comparison with corrected *p*-value > 0.05 was considered to be not statistically significant and denoted by “ns” (not significant).


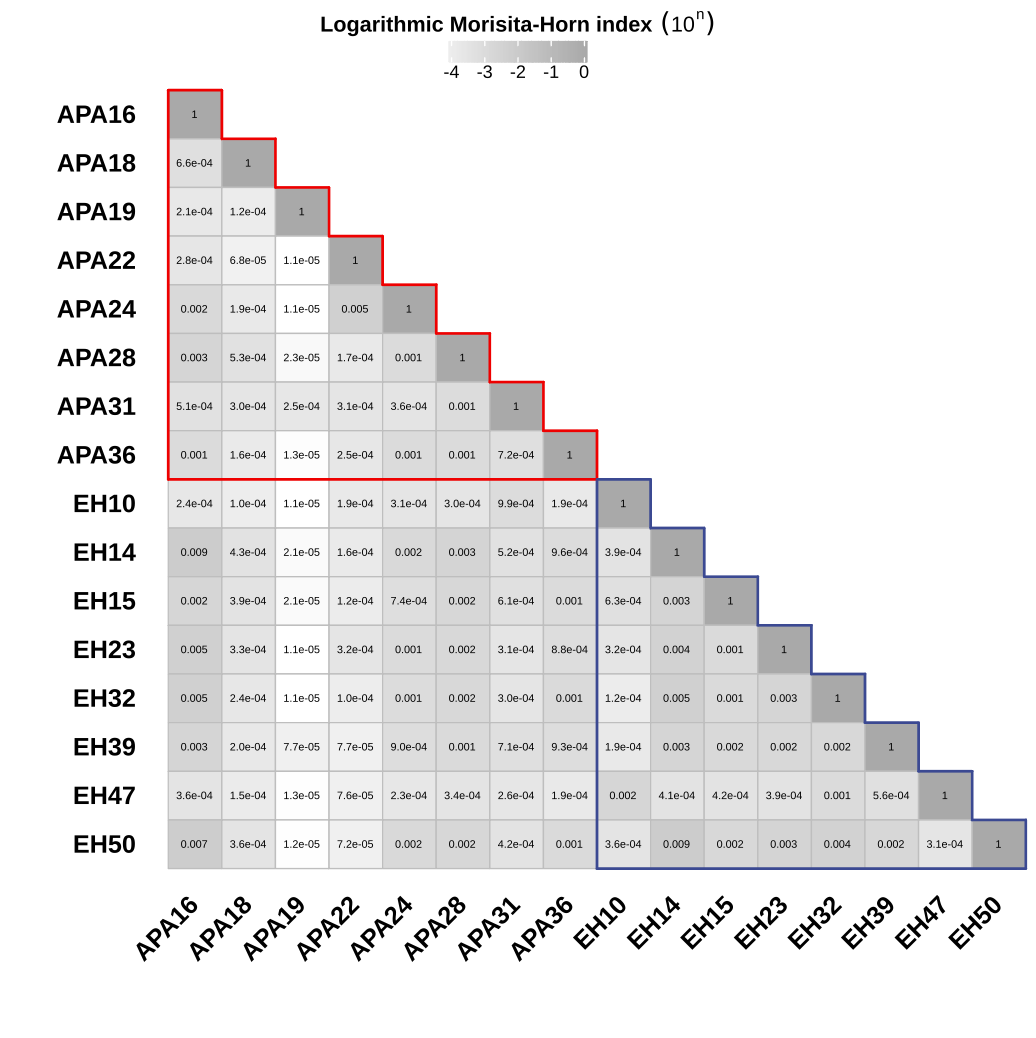


**Figure S2.** The Morisita-Horn (MH) similarity between TCRβ repertoire of patients. The intragroup (EH vs. EH or APA vs. APA) and intergroup (EH vs. APA) MH indices were illustrated in the heat map. The intragroup comparison was highlighted by red and blue borders for MH similarities between APA patients and between EH individuals, respectively.


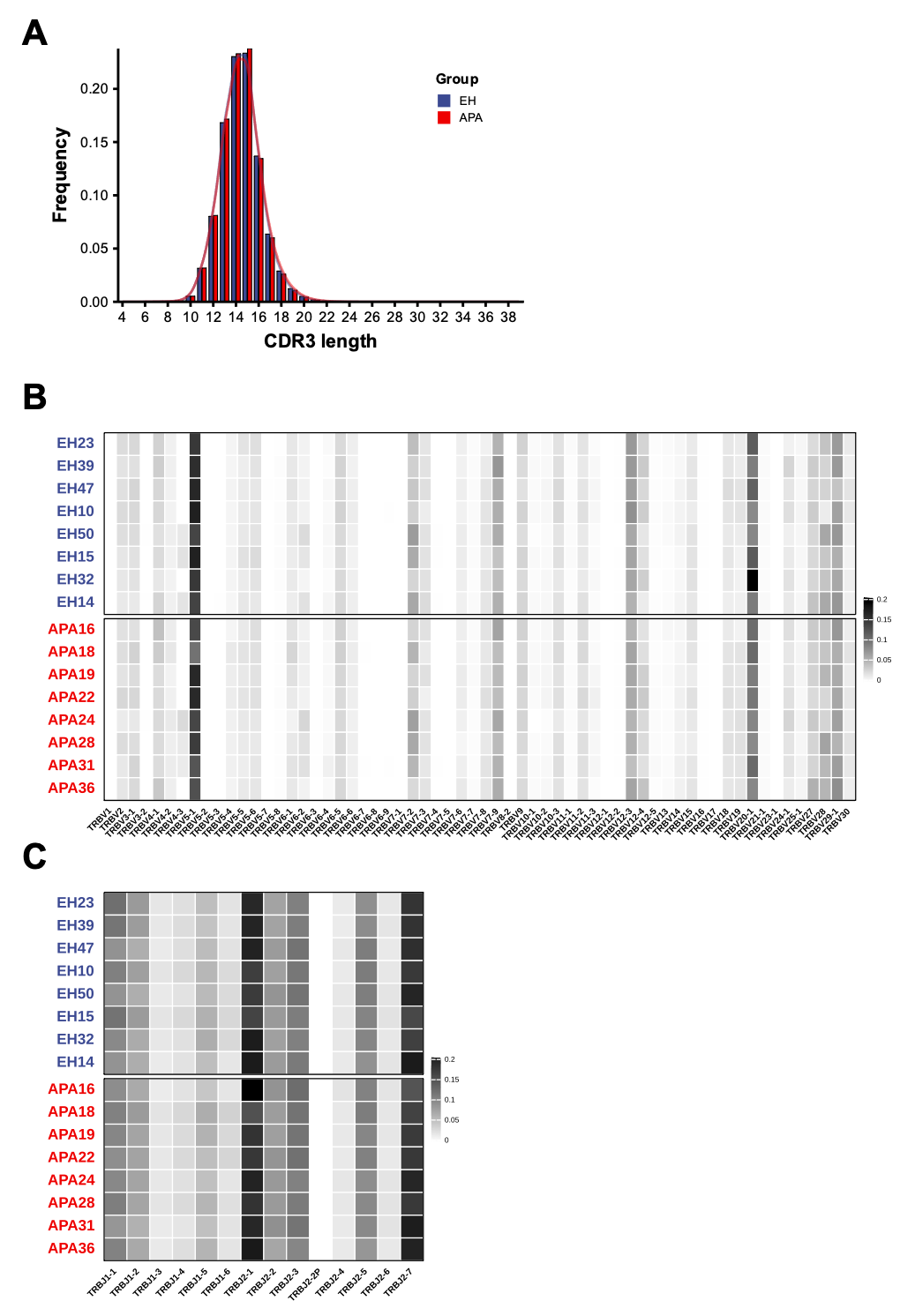


**Figure S3.** The CDR3 length distribution and V/J gene usage in TCRβ repertoires of APA and EH patients. (**A**) The distribution of CDR3 lengths was depicted on the basis of unique TCRβ clonotypes in EH (blue) and APA (red) group. Each bar represented the total counts of specific CDR3 length across TCRβ repertoires of EH or APA patients. Only those of TCRβ clonotypes with CDR3 length less than or equal to 40 amino acids were included. The *TRBV* (**B**) and *TRBJ* (**C**) gene usage of unique TCRβ clonotypes in EH (labeled by blue) and APA (labeled by red) patients was illustrated. The gradient color represented the proportion of each V or J gene fragment within the TCRβ repertoire.


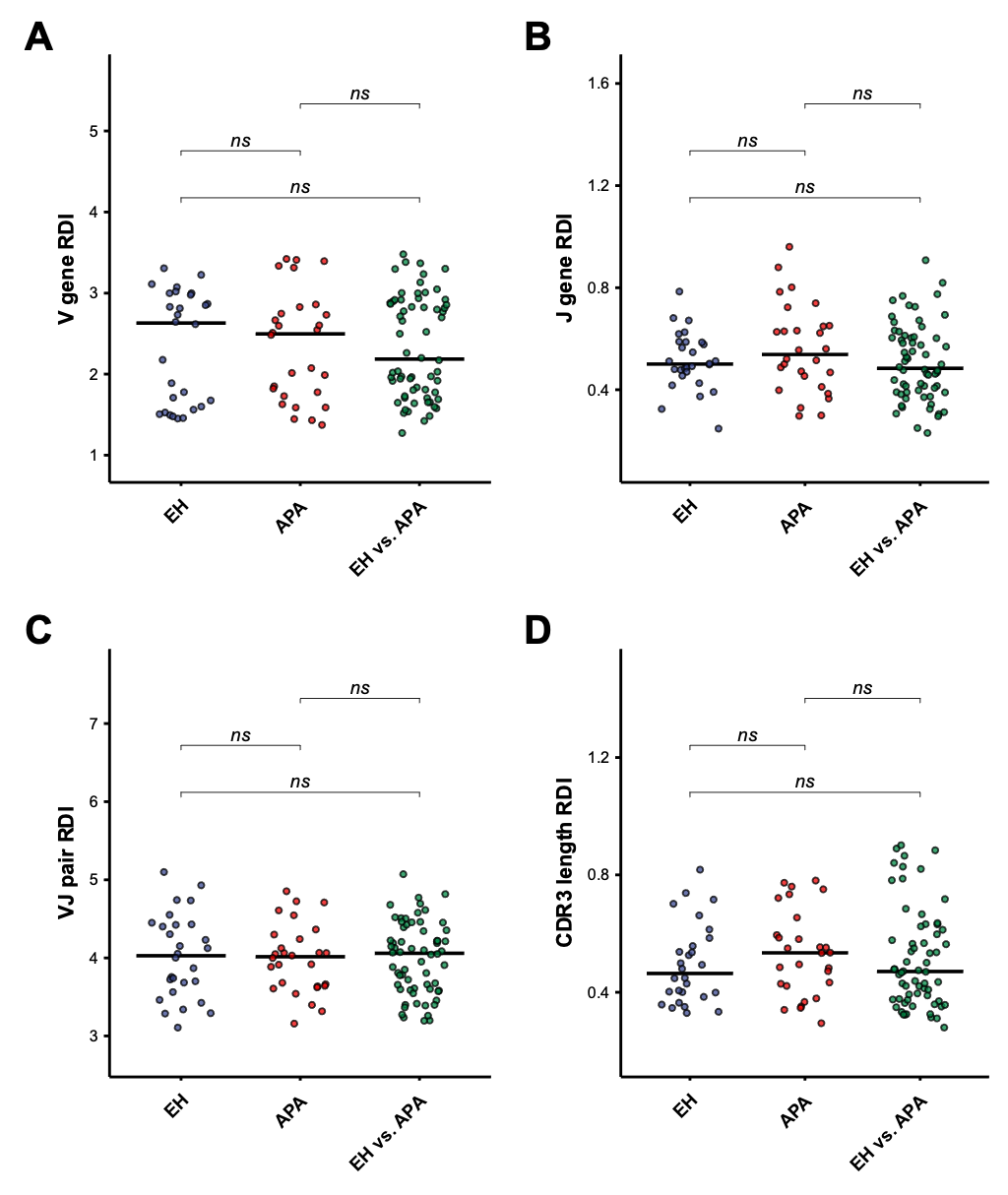


**Figure S4.** The dissimilarity of V and J gene usage, V-J gene pairing and CDR3 length profiles between APA and/or EH patients. The repertoire dissimilarity index (RDI) was calculated for V gene (**A**) and J gene (**B**) segment, V-J gene pair (**C**) and CDR3 length (**D**). The intragroup (EH vs. EH and APA vs. APA) and intergroup (EH vs. APA) RDI values were compared. The horizontal line represented the median. The FDR-adjusted two-sided *p*-values were shown from the Wilcoxon rank-sum test. The results of comparison with corrected *p*-value > 0.05 was considered to be not statistically significant and denoted by “ns” (not significant).
